# The road to success of coagulase-negative staphylococci: clinical significance of small colony variants and their pathogenic role in persistent infections

**DOI:** 10.1007/s10096-021-04315-1

**Published:** 2021-07-23

**Authors:** Agnieszka Bogut, Agnieszka Magryś

**Affiliations:** Chair and Department of Medical Microbiology, Medical University of Lublin, ul. Chodźki 1, 20-093 Lublin, Poland

**Keywords:** *Staphylococcus epidermidis*, Coagulase-negative staphylococci, Small colony variants, Device-related infections, CoNS pathogenesis, Intracellular persistence

## Abstract

Bacterial small colony variants represent an important aspect of bacterial variability. They are naturally occurring microbial subpopulations with distinctive phenotypic and pathogenic traits, reported for many clinically important bacteria. In clinical terms, SCVs tend to be associated with persistence in host cells and tissues and are less susceptible to antibiotics than their wild-type (WT) counterparts. The increased tendency of SCVs to reside intracellularly where they are protected against the host immune responses and antimicrobial drugs is one of the crucial aspects linking SCVs to recurrent or chronic infections, which are difficult to treat. An important aspect of the SCV ability to persist in the host is the quiescent metabolic state, reduced immune response and expression a changed pattern of virulence factors, including a reduced expression of exotoxins and an increased expression of adhesins facilitating host cell uptake. The purpose of this review is to describe in greater detail the currently available data regarding CoNS SCV and, in particular, their clinical significance and possible mechanisms by which SCVs contribute to the pathogenesis of the chronic infections. It should be emphasized that in spite of an increasing clinical significance of this group of staphylococci, the number of studies unraveling the mechanisms of CoNS SCVs formation and their impact on the course of the infectious process is still scarce, lagging behind the studies on *S. aureus* SCVs.

## Introduction

Variability within bacterial populations has become a concept gaining an increasing interest in the field of microbiology due to its correlation with the pathogenesis of infectious diseases. It may contribute to microbial virulence, persistence, and consequent survivability [1].

It has been reported that even within a genetically clonal bacterial population, a variety of phenotypic variants may arise. These, in turn, can be referred to as alternative microbial lifestyles increasing the chance to survive under unfavorable environmental conditions exemplified by host immune responses and antibiotic selective pressure. Phenotypic switches include formation of biofilms, persister cells, and small colony variants (SCVs) [2]. This strategy has undoubtedly paved the way to “success” for medically important microorganisms, especially those, whose inherent pathogenic potential is low.

In spite of the fact that their default status is permanent members of the human skin and mucous membranes microbiota, coagulase-negative staphylococci (CoNS) possess a sufficient armamentarium of factors and strategies to act as opportunistic pathogens, which are determined by specific host conditions as well as by specific species- and strain-dependent features [3]. The incidence of CoNS has steadily increased over the past decades in parallel to the advancement in medicine, especially in regard to the utilization of foreign body devices [4]. Although CoNS represent a heterogeneous group, ranging from true nonpathogenic to facultative pathogenic species with low, medium, or even high virulence potential, with the latter exemplified by *Staphylococcus lugdunensis* (resembling *S. aureus* in some regards) [3], they can be tied by common threads involved in transition to a pathogenic interaction with the host. They include not only well-recognized remarkable adhesive properties and high antibiotic resistance rates but also phenotypic variation that enables rapid adaptation of physiology for survival [1, 3, 5]. Phenotypic variation is involved in bacterial ability to occupy novel niches, but also to sustain within the host and even to modulate the course of an infection [3].

SCVs represent an important aspect of bacterial variability. They are naturally occurring microbial subpopulations with distinctive phenotypic and pathogenic traits, reported for many clinically important bacteria [6].

Phenotypically, SCVs can be defined as a slow-growing phenotype demonstrating atypical colony morphology and unusual biochemical characteristics. These features make SCVs a challenge for the correct isolation and identification in routine laboratories [6, 7].

In clinical terms, SCVs tend to be associated with persistence in host cells and tissues and are less susceptible to antibiotics than their wild-type (WT) counterparts [6, 8, 9]. The increased tendency of SCVs to reside intracellularly where they are protected against the host immune responses and antimicrobial drugs is one of the crucial aspects linking SCVs to recurrent or chronic infections, which are difficult to treat [6]. Moreover, the instability of SCVs reflected by their ability to revert to the WT phenotype when leaving their host cells would also provide a mechanism for relapsing, virulent infections [10, 11]. On the other hand, it should be noted that non-stable SCVs may switch to stable SCVs during prolonged survival within host cells, under certain antimicrobial treatments or intracellular stress conditions [12]. Finally, an important aspect of the SCV ability to persist in the host is the quiescent metabolic state, reduced immune response and expression a changed pattern of virulence factors, including a reduced expression of exotoxins and an increased expression of adhesins facilitating host cell uptake [13–15].

The purpose of this review is to describe in a greater detail the currently available data regarding CoNS SCV and, in particular, their clinical significance and possible mechanisms by which SCVs contribute to the pathogenesis of chronic infections. It should be emphasized that in spite of an increasing clinical significance of this group of staphylococci, the number of studies unraveling the mechanisms of CoNS SCVs formation and their impact on the course of the infectious process is still scarce, lagging behind the studies on *S. aureus* SCVs.

## Review methodology

This review was exclusively based on thorough literature review on the characteristics of the SCV phenotype and diagnostic and therapeutic challenges as well as interaction of the CoNS SCVs with the host cells affecting intracellular survival. The Boolean search for relevant literatures was performed in PubMed, Scopus, and Google Scholar, using the following precise keywords: *Staphylococcus epidermidis*; coagulase-negative staphylococci; small colony variants; SCV selection; auxotrophism; device-related infections; laboratory diagnosis; drug-susceptibility testing; antibiotics resistance; CoNS pathogenesis; virulence factors in CoNS; intracellular persistence; and combinations thereof.

The search was limited to studies published in the English language and available online as full text. Bibliographies were hand-searched for secondary references. All the papers that relate to CoNS SCV clinical and diagnostic relevance and mechanisms of its pathogenesis were included in the study.

### Key facts on the SCV phenotype

#### How does the SCV phenotype arise?

The SCV morphotype can be considered a general strategy for bacterial survival. The phenotypic switching between WT and SCVs has been reported to constitute a part of the bacterial exponential-phase growth without any selective pressure as shown in the study of Edwards et al. [8]. The authors demonstrated that *S. aureus* phenotype switching occurs via a constitutive mechanism dependent upon bacterial replication that generates a dynamic, antibiotic-resistant subpopulation able to revert to the parental phenotype.

On the other hand, however, environmental sub-optimal conditions can also lead to induction and selection for staphylococcal SCVs. In this regard, the SCV phenotype should be perceived as a microbial strategy aimed at survival and long-term persistence [11].

Onyango et al. [1] investigated formation of the SCV phenotype by both *S. aureus* and CoNS including *S. epidermidis* and *S. lugdunensis* following exposure of the WT clinical isolates to low temperature, antibiotic (penicillin and vancomycin) stress, pH stress, and osmotic challenge. They found that prolonged exposure to all of the treatments led to a subsequent formation of the SCV phenotype. Perez and Patel [16, 17] studied the reference *S. epidermidis* strain RP62A as well as two *S. epidermidis* isolates from prosthetic joint infections and demonstrated not only that a low pH favors *S. epidermidis* SCV formation, but also that the intracellular environment has an analogous effect, as it has been previously shown for *S. aureus* [10, 18, 19]. The intracellular selective pressure towards SCV formation can possibly be provided by host cationic antimicrobial peptides [14, 20].

Other stimuli known to favor staphylococcal SCV formation include prolonged growth under nutrient limiting conditions [21], oxidative stress [2, 9, 22], or exposure to disinfectants [23].

Chronic antibiotic exposure has been regarded as one of the key factors involved in the formation of SCVs with a striking example of cystic fibrosis patients who receive large quantities of antibiotics [24–26] or orthopedic patients who experience chronic exposure to aminoglycosides including the surgical placement of gentamicin beads in the infected bones [27]. In the latter group, staphylococcal SCVs including those represented by CoNS are observed with high regularity. The majority of studies reporting the isolation of CoNS SCVs from clinical infections have been associated with medical device-associated infections [28–36]. Tande et al. [31], for example, investigated clinical characteristics and outcomes of PJIs caused by SCV staphylococci and revealed that subjects with SCVs were more likely to have received prior surgical and chronic antimicrobial therapy for their infection. Interestingly, the authors observed SCVs with similar frequency among *S. aureus* strains and various species of CoNS.

#### Mechanisms underlying the selection of SCVs

Mechanisms underlying the selection of SCVs include genetic changes and regulatory mechanisms leading to a lifestyle change accompanied by a profound multifaceted conversion of the microorganism’s metabolism [11]. It has been suggested that at early stages of the infectious process, the first SCVs are rapidly formed via regulatory mechanisms, which enables the bacteria to dynamically respond to changing environmental conditions. As a result, infecting pathogens can hide within host cells and escape both the immune response and the action of antimicrobial drugs. As the infectious process progresses, due to the inability of host defenses and antibiotic treatment to eliminate microbial intruders, chronically infecting bacteria may apply further strategies to form permanent SCVs, such as defined mutations [11].

Many clinical staphylococcal SCVs can be tied by a common thread, which are alterations in electron transport. Defects in the electron transport lead to a drastically reduced amount of ATP available for the cell wall biosynthesis which, consequently, contributes to a lower growth rate or decreased pigment formation [6, 11]. Moreover, a decrease in the electron transport activity may account for bacterial resistance to several antibiotics as well as provide a mechanism for persisting within host cells [37].

Electron transport deficiency is typically observed for SCVs which demonstrate hemin, menadione, and thymidine auxotrophism [14]. Auxotrophism defined as the inability of the microorganism to synthesize specific compounds which makes it dependent on their external sources [15], represents one of the most important and most common metabolic changes observed in SCVs and has been linked to several underlying mutations.

The SCV phenotype can be induced by disruptions of the metabolic pathways conferred either by mutations in the *thyA* gene encoding for thymidine synthetase mediating the conversion of uracil to thymidine which is essential for DNA synthesis or by mutations in menadione and hemin production pathways. Auxotrophy for menadione and hemin makes the bacteria unable to synthetize menaquinone and cytochromes, respectively, which are important components of the electron-transport system [6, 38, 39]. The *thyA* mutants have been grouped with electron transport-deficient bacteria due to the fact that this mutation results in a reduced level of ClpC (caseinolytic protease), which is required for the expression of aconitase. Reduced aconitase activity decreases Krebs cycle activity, which is linked to the downregulation of electron transport chain biosynthetic enzyme expression [12, 14, 40].

The underlying mutations have been identified in several genes encoding for enzymes involved in the biosynthesis of menadione and hemin including *menB*, *menD* and recently identified in clinical *S. aureus* SCVs *menC*, *menE*, *menF* as well as *hemB*, *hemH*, *hemA*, and *hemG* genes [22, 38, 41–46]. For clinically derived SCVs, only mutations of the *hemG*, *menB*, *menC*, *menE*, *menF*, and *thyA* genes have been identified, and they were reported for *S. aureus* exclusively [22, 38, 41–46].

It should be emphasized that electron transport deficient SCVs have been characterized by significant modifications of their central metabolic processes. Previous studies reported downregulation of the Krebs cycle and the global regulator *agr* [40, 45, 47–50].

Agr, generally conserved within the staphylococci, is the quorum sensing two component system (TCS) that upregulates the expression of secreted toxins and enzymes contributing to the development of inflammation and host cell destruction. Quorum-sensing is population density-dependent and environment-dependent gene regulation that occurs through cell–cell communication. It has been considered one of the most important regulatory mechanisms that ensure timely adaptation of staphylococcal physiology to the environment [11, 51]. Quorum sensing is controlled by the secreted Agr inducing peptide (AIP) which upregulates the *agr* expression in surrounding staphylococcal cells. The *agr* system is highly upregulated in acute infections and, as mentioned above, downregulated in persisting SCV-like bacteria due to intracellular nutrient restriction and the presence of host cationic peptides. It has been observed that respiration-defective staphylococcal SCVs consistently show reduced levels the effector molecule — RNA III — a non-coding regulatory RNA encoded by the *hld* gene which is directly regulated by AgrA [11, 12, 43, 44, 52, 53]. Changes in RNAIII observed in persistent infections associated with SCVs can occur through RNA degrasome, production of small RNAs, and toxin-antitoxin systems which has been extensively discussed by Proctor et al. [14].

On the other hand, downregulation of the *agr* system has been coupled with upregulation of a stress-related transcription factor SigB. High levels of SigB have been found in clinical SCVs isolated from patients with cystic fibrosis and osteomyelitis. This factor has been reported to regulate the switch to dormancy in *S. aureus* in opposition to the *agr* and its regulation of virulence. It promotes biofilm formation and the expression of adhesins. SigB activity has been shown to allow SCVs to persist intracellularly within human endothelium. This also is associated with the increase in expression of FnBPs which then contributes to the biofilm formation, adhesion, and intracellular persistence. SigB is also required for intracellular replication of SCVs. SigB and subsequent *agr* repression is required for the SCV formation in response to the aminoglycoside stress [2, 11, 54–57].

In SCVs, other negative regulators of Agr also exhibit increased expression including ArlRS, CodY, SrrAB, VraR, and RsaE. Some positive regulators are inhibited (MgrA) or exhibit reduced expression (CyoE and SarA). Exceptions to the pattern of negative regulation of Agr are SarA and SarU, which were shown to be highly expressed in *hemB* and *menD* mutants and other SCVs [44, 58], but they compose a positive regulator of Agr. However, SarA expression is downregulated in thymidine mutants [59]. Overall, RNAIII production is reduced as a result of the regulatory balance in SCVs, which decreases its production [12].

Electron-deficient SCVs also demonstrate increased expression of the arginine deiminase (AD) pathway. In the presence of arginine, the AD pathway allows staphylococci to grow anaerobically. It has been suggested that SCVs unable to use the citric acid cycle and electron transport employ this pathway to compensate for the loss [43]. The AD pathway has also been linked to the intracellular persistence of SCVs. The AD pathway can be used not only to produce ATP but also, through ammonia production, to counteract the acidic environment that prevails intracellularly [43, 44, 60].

It has been reported that hemin- and menadione-dependent SCVs arise after treatment with aminoglycosides [61]. The resultant SCVs are resistant to this group of antimicrobials as well as to other cationic substrates including defensins or antimicrobial peptides such as lactoferrin B [7]. The resistance mechanism has been linked to a decrease in the cell membrane potential compared to the WT cells which prevents the uptake of the positively charged molecules [6, 7, 9]. It has also been revealed that staphylococcal strains carrying a *fusE* mutation grow as SCVs and demonstrate resistance to fusidic acid. They can be selected with either fusidic acid or aminoglycosides. Moreover, FusE SCVs harbor mutations in a *rplF* gene encoding ribosomal protein L6 and contain mutations in genes required for hemin and menadione biosynthesis as well [41, 62].

Hemin and menadione auxotrophy in clinical CoNS SCVs has been reported by several authors, and it has been correlated with aminoglycoside resistance in the majority of isolates [29, 31, 32, 34, 36].

Thymidine-dependent SCVs emerge after prolonged treatment with trimethoprim-sulfamethoxazole, and they have been mostly related to chronic airway infections in cystic fibrosis (CF) patients. These antimicrobial agents inhibit the tetrahydrofolic acid synthesis, which serves as a cofactor for thymidylate synthetase (*thyA*). Thymidine-dependent SCVs can survive only in the presence of exogenous thymidine provided in infected tissues due to the cell degradation and the presence of pus since they carry mutations in the *thyA* gen [6]. To the best of the knowledge of the authors of this publication, there has been only one report of a clinical *S. epidermidis* SCV from PJI with thymidine dependence detected [29].

The importance of defects in the electron transport was first shown by von Eiff et al. [63] who generated a stable electron-transport mutant of *S. aureus* by interruption of the *hemB* gene encoding for an enzyme involved in biosynthesis of the porphyrin ring that is present in the haem prosthetic group. The authors found that the mutant showed typical characteristics of clinical SCVs, such as slow growth, decreased pigment formation, low coagulase activity, reduced hemolytic activity, and resistance to aminoglycosides. Additionally, the mutant was able to persist within cultured endothelial cells due to decreased alpha-toxin production. Northern and Western blot analyses showed that expression of alpha-toxin and that of protein A were markedly reduced, at both the mRNA and the protein level. The SCV phenotype of the *hemB* mutant was reversed by growth with hemin or by complementation with an intact *hemB*. Vaudaux et al. [52] reported that the SCV *hemB* mutant of *S. aureus* displayed a significantly higher adhesion to fibrinogen and fibronectin compared to its isogenic WT parent strain which was correlated with an increased surface display of the adhesins. These observations were supported by an increased expression of the *clfA* and *fnb* genes encoding for fibrinogen- and fibronectin-binding proteins, respectively. The increased surface display of these proteins occurred independently of the *agr* system and was also suggested to be involved in an efficient internalization of *hemB* mutants by embryonic kidney cells.

Similar investigation based on the generation of a stable *S. epidermidis* mutant displaying the SCV phenotype by inactivation of the *hemB* gene was conducted by Al-Laham et al. [64]. This gene reveals 89.2% DNA sequence similarity to the corresponding *hemB* gene in *S. aureus* and 96% identity on the protein level. In the study, the *S. epidermidis hemB* mutant showed properties similar to those observed for SCVs isolated from clinical specimens and to the *S. aureus hemB* mutant generated previously by von Eiff et al. [63]. They included pinpoint colonies growing at a slow rate and decreased susceptibility to aminoglycosides. Of note, the mutant expressed larger amounts of the polysaccharide intercellular adhesin (PIA) — the major component of the staphylococcal biofilm matrix — than the corresponding WT strain and the complemented mutant. The authors linked the inability of the *hemB* mutant to use the respiratory chain, including the oxygen or nitrate as a terminal electron acceptor to a decreasing activity of the Krebs cycle, increased acetate level, and the resultant activation of the *ica* operon. The authors also found evidence that the alternative sigma factor σ^B^ is upregulated in the *hemB S. epidermidis* mutant, which plays an important role in the control of the expression of the *icaADBC* operon (responsible for the PIA synthesis) in response to various stimuli.

Nevertheless, there have been discrepancies between the in vitro and in vivo studies regarding increased biofilm formation by CoNS SCVs. Sander et al. [65] developed a murine model of catheter colonization and abscess formation due to *S. epidermidis* with normal and SCV phenotypes and found that that the pathogenic process associated with different phenotypes of *S. epidermidis* is mouse strain dependent. The authors reported that the *hemB* mutant of *S. epidermidis* did not colonize catheters at amount comparable to that of the WT with the exception of the CD-1 mouse strain and at a dose of 10^8^ bacteria in BALB/c mice. It corresponded with the observation that abscesses associated with *S. epidermidis* SCV infection were smaller than those formed by the WT. In general, results obtained by Sander et al. did not confirm the previously reported findings on the more efficient biofilm production by *S. epidermidis hemB* mutant [64]. The authors speculated that a combination of defects in electron transport, resulting in growth retardation could have led to a reduced biofilm formation in vivo and a subsequent reduced survival in mice.

Studies on clinical CoNS SCVs originating from human infections have also brought conflicting results. Baddour et al. [66] showed that *S. epidermidis* SCVs associated with an infection of endocardial pacemaker electrodes were strong biofilm (slime) formers. High biofilm formation levels for the SCV strain of *S. epidermidis* auxotrophic for hemin, menadione, and thymidine involved in PJI were also reported by Ortega-Peña et al. [28]. Nevertheless, Maduka-Ezeh et al. [33] who analyzed 11 *S. epidermidis* SCVs recovered from PJIs, among which none of the isolates showed the above mentioned types of auxotrophies, found that only two strains were proficient biofilm formers and did not observe a significantly enhanced biofilm-forming capacity of PJI-associated *S. epidermidis* SCVs compared to the normal phenotype isolates. Similarly, Bogut et al. [32], who investigated hemin-dependent *S. epidermidis* (including one *S. epidermidis* strain with a double auxotrophy for hemin and menadione) and *S. warneri* SCV isolates from PJIs, detected the *icaADBC* operon mediating the PIA-dependent biofilm formation in only 1 out of 8 isolates. The *ica*-positive isolate was the only strong biofilm producer among the SCVs. The remaining SCVs were capable of the moderate, presumably protein-mediated (due to the protease sensitivity) biofilm production, and, interestingly, they did not outcompete their WT counterparts in this regard.

In addition to electron transport deficiency, several other pathways leading to the SCV development when ATP levels are not reduced by interruption of electron transport have been reported. These SCVs also cause chronic infections and have low RNAIII levels [12]. The examples include *S. aureus* SCVs with mutations in lipid biosynthesis genes. Hitherto, the fatty acid metabolism-linked genes *accC*, *accD*, *fabF*, *fabI* (eventually combined with *fabD*), *plsX* and *ecf* (energy-coupling factor) module have been associated with the phenotype switch of fatty acid-auxotrophic SCVs [67].

Another type of auxotrophism identified among SCVs is carbon dioxide dependency. It has been reported both in *S. aureus* and in *S. epidermidis* SCVs associated with clinical infections. *S. aureus* SCVs reported by Gómez-González et al. [68] were isolated from a variety of clinical infections including catheter-related bacteremia, deep, wound, and respiratory infections as well as from cases of nasal colonization. *S. epidermidis* CO_2_-dependent SCVs were cultured from patients suffering from prosthetic-joint infections [31, 33]. CO_2_ auxotrophs grow as normal-sized colonies on the plates incubated in 5% CO_2_ and as pinpoint SCVs on the plates incubated in air [33]. Unfortunately, the underlying mechanism of CO_2_ dependence remains unknown.

There have also been other genetic mechanisms considered important for the formation of SCVs. Gao reported a point mutation in the *relA* gene that resulted in a reduced RelA hydrolase activity and a consequent permanent activation of the stringent stress response due to the accumulation of its effector compound — a small signalling molecule called (p)ppGpp. This mechanism was described for SCVs isolated from a patient with chronic MRSA bacteremia, treated with various antibiotics [69].

Prolonged nutrient starvation and stressed conditions have in turn been coupled with single nucleotide polymorphisms or insertions-deletions potentially implicated with the switch to SCV [21]. Bui et al. revealed a new type of a stable *S. aureus* SCV of clinical origin characterized by mutations in *mgrA* (a global regulator) and *rsbU* (a phosphoserine phosphatase within the regulatory pathway of the sigma factor SigB). MgrA positively affects the expression of capsular polysaccharide, alpha-toxin, leukocidins, coagulase, protein A – downregulated in SCVs; the loss of *mgrA* enhances autolysis, invasion of HeLa cells, increases biofilm formation and expression of surface proteins including microbial surface components recognizing adhesive matrix molecules (MSCRAMMs) [2, 21].

Moreover, stressed conditions cause damage to bacterial DNA and the resultant SOS response creates mismatch repairs followed by increased rate of mutation associated with a higher frequency of SCV formation. Higher mutation rate is favoring the SCV formation in response to environmental stimuli including sub-inhibitory concentration of antibiotics such as fluoroquinolones, aminoglycosides [9, 22]. Hypermutability resulting from mutations in genes responsible for the DNA repair has also been detected in thymidine-dependent *S. aureus* SCVs isolated from CF patients. Moreover, hypermutability favors the acquisition of antibiotic resistance and facilitates bacterial adaptation during long-term persistence [39].

#### Clinical significance of CoNS SCVs

The recovery of CoNS SCVs and their involvement in device-related infections has a rising tendency however, still lagging behind the studies on *S. aureus* SCVs. Hitherto, CoNS SCVs have been associated with foreign body-related infections including pacemaker-related infections [34, 36, 66], catheter-associated infections [35], and PJIs [28–33].

To date, etiology of these infections has included *S. epidermidis* [28, 31–33, 35, 36], *S. warneri* [32], *S. capitis* [31, 36], and *S. lugdunensis* [29–31, 34]. Summary of the publications reporting isolation of CoNS SCVs from human infections is provided in Table 1.

**Table 1:** Studies and case reports on CoNS SCVs

Type of infection	Species	Clinical details	Auxotrophy	WT	Reference
Pacemaker-related bloodstream infection	*S. epidermidis* *S. capitis*	Heterogenous culture, biochemical identification impossible, identity confirmed by the sequence analysis of a portion of 16S rRNA gene, isolates clonal by PFGE, susceptible to antibiotics tested Mostly growing as pinpoint colonies; upon subculture — some colonies grew rapidly, isolates clonal by PFGE; the pacemaker removed — the SCV culture result positive, susceptible to antibiotics tested	Hemin dependency Hemin dependency		von Eiff et al. 1999 [36]
Pacemaker-related bloodstream infection	*S. lugdunensis*	SCV cultured from thrombotic material scraped from the pacemaker leads; culture result positive after 48-h incubation; mixed population; colony variations persisting after single-colony subcultures Biochemical identification (API ID 32 Staph system BioMerieux), confirmed by 16S rRNA gene sequencing, all isolates clonal by PFGE; large colony morphotype susceptible to all antimicrobial agents in the Vitek (BioMerieux) test card; susceptibility to penicillin and oxacillin confirmed for all colony variants by E-test; VITEK system failed to perform antibiotic susceptibility testing of SCV	Hemin dependency	Present	Seifert et al. 2005 [34]
Prosthetic joint infections	*S. epidermidis*	11 isolates cultured from explanted prosthetic joints using sonication; identification by MALDI-TOF, 16S ribosomal RNA gene sequencing; susceptibility testing with agar dilution, disc diffusion and E-tests — susceptibilities read at 48 h to accommodate the slow growth of SCVs; 9/11 were *mecA* positive but phenotypic oxacillin resistance in the 8/9; 5/11 aminoglycoside resistant 3/11 isolates very stable, 3/11 unstable, 5/11 very unstable Three iSCVs demonstrated a “fluctuating phenotype” — following reversion to WT, further passage led to the reappearance of the SCV phenotype 2/11 isolates were proficient biofilm producers at 48 h, 7/11 — poor biofilm formers, 2/11 — intermediate biofilm formers; no difference with regard to cell wall thickness, presence of ghost cells, heterogeneity of cell shape and size compared to WTs	3/11 isolates CO_2_ auxotrophic	3 SCV-WTs pairs (1 indistinguishable by PFGE, 2 closely related, 1 possibly related)	Maduka-Ezeh et al. 2012 [33]
Prosthetic joint infections	*S. epidermidis* *S. warneri*	8 SCV isolates obtained from 6/31 culture-positive patients with revision of total hip prostheses (isolation from the sonicate fluid/synovial fluid/periprosthetic tissues); identification by a commercially available PCR assay followed by hybridization; 2 patients with 2 phenotypically distinct SCV isolates and co-cultured WTs, one SCV monoculture without a WT counterpart; 7/8 isolates *mecA* positive but phenotypic cefoxitin resistance in the 6/7; 7/8 isolates aminoglycoside resistant; 1/8 TMP-SXT resistant 1 isolate *icaADBC*-positive and producing strong biofilm; the remaining isolates *ica-*negative, moderate producers of trypsin-sensitive biofilm SCVs did not overwhelm/outweigh their WT counterparts biofilm forming ability	Hemin dependency in all SCVs; 1 isolate with combined auxotrophy (hemin + menadione)	5 indistinguishable SCV/WT pairs, 1 related pair (rep-PCR)	Bogut et al. 2014 [32]
Prosthetic joint infections	*S. epidermidis* *S. capitis/caprae* *S. lugdunensis*	Isolation of SCVs from the sonicate fluid culture 38/113 patients SCV-positive Species identification by MALDI-TOF 22/38 isolates *S. epidermidis* 10/38 *S. aureus* 2/38 *S. capitis/caprae* 3/38 *S. lugdunensis* 5/38 subjects had only SCV isolated without a co-isolated WT strain Antibiotic susceptibility testing on MHA supplemented with 5% sheep blood due to poor growth (in 11 subjects) 22/42 isolates: oxacillin resistant 6/42: gentamicin resistant	Hemin dependency 1/42 isolates CO_2_ dependence: 8/42 isolates CO_2_ and menadione dependence: 1/42 isolates	19/38 subjects: *S. epidermidis* SCV-WT pairs; 3/38 *S. lugdunensis* SCV-WT pairs; 2/38 *S. capitis/caprae* SCV-WT pairs; 16 pairs indistinguishable 3 pairs closely related (PFGE)	Tande
Prosthetic joint infections	*S. lugdunensis*	WT and SCV in mixture, cultured from tissue samples and sonicate fluid Identification by MALDI-TOF; similar antibiotic susceptibility profiles of WT and SCV	Unknown	SCV-WT indistinguishable (MALDI-TOF)	Askar
Prosthetic joint infections	*S. lugdunensis*	Prosthetic knee infection that relapsed 1 year later with the same strain that changed from antibiotic susceptible WT to antibiotic resistant SCV First WT isolation from the joint fluid Relapse manifested as a sinus tract with purulent discharge. SCV cultured after 3 days of incubation in the sonicate fluid, followed by periprosthetic biopsies and synovial tissue cultures Identification by MALDI-TOF Antimicrobial susceptibility testing showed that the SCV strain was resistant to fluoroquinolones and amikacin	Unknown	WT and SCV belonged to ST1 and virulence type 1 (MLST and MVLST)	Argemi
Prosthetic joint infections	*S. epidermidis*	SCV isolated from the relapse of the PJI (bone, prosthesis, soft tissue), stable, strong biofilm production	Hemin, menadione, thymidine dependency		Ortega-Peña et al
Catheter-associated bloodstream infection	*S. epidermidis*	SCV isolated from blood cultures collected from a neutropenic patient with leukemia Unstable phenotype, quickly reverting to the WT. Biochemical identification confirmed by 16S rRNA sequencing. Susceptibility testing performed using broth microdilution, detection of PBP2’, E-tests using the M-H agar with 5% sheep blood WT and SCV — resistant to methicillin, tobramycin, co-trimoxazole, ciprofloxacin, levofloxacin WT — teicoplanin sensitive SCV — teicoplanin resistant	Unknown	WT and SCV clonal (PFGE)	Adler

*MALDI-TOF* matrix-assisted light desorption ionization-time of flight mass spectrometry

All patients who developed pacemaker-related infections associated with CoNS SCVs had a long history of having their pacemakers in place. All of them experienced preceding manipulations of the systems including replacement of the pacemaker [66], tooth extraction [36], or replacement of the battery [34]. In spite of an initial good response to treatment, the patients presented with relapsing episodes of fever and chills, and their antimicrobial treatment was extended for up to several months. SCVs were cultured form the surface of the device including scrapings of the thrombotic material from pacemaker leads [34] and from the bloodstream [36]. In the majority of cases, the recovery was possible only after a total removal of the device [34, 36]. Staphylococcal SCV species involved in the pathogenesis of the above mentioned infections included *S. epidermidis*, *S. capitis*, and *S. lugdunensis*.

Adler et al. [35] reported a case of infection with a teicoplanin-resistant SCV of *S. epidermidis* (clonal with its WT which was teicoplanin sensitive) isolated from blood cultures of a patient with acute leukemia and therapy-induced neutropenia who was treated with vancomycin for a catheter-associated bloodstream infection. Despite removal of the catheter and adequate antibiotic therapy, the infection did not clear, and the patient died 20 days after continuous antibiotic therapy as a result of non-responding clinical sepsis.

The association of CoNS SCVs with PJI has been reported in studies investigating SCVs obtained both from patients who had explanted prosthetic joints due to presumed infection (based on the presence of a sinus tract communicating with the prosthesis, gross purulence noted at surgery, and /or acute inflammation on intraoperative frozen section histology) and clinically aseptic implant failure. CoNS SCVs grew in the sonicate fluid cultures [29–33] and/or periprosthetic tissue samples [28, 32].

Clinical history of patients including their prior antibiotic treatment has not been available in all of the published studies. However, data provided in the study by Tande et al. [31] who identified PJIs associated with staphylococcal SCVs in 38 (34%) out of 113 patients indicate that subjects with SCVs were more likely to have been exposed to chronic antibiotic therapy prior to surgery, have had prior surgery for PJI, have had a longer duration of symptoms, and have had a longer time since reimplantation compared to those infected with WT bacteria only. The predominant SCV species was *S. epidermidis* (57.9%), followed by *S. aureus* (26.3%), *S. lugdunensis* (7.9%), and *S. capitis/caprae* (5.3%). Over a median follow-up of 30.6 months, 24% of patients with SCVs and 32% infected with WT only experienced treatment failure defined as subsequent revision surgery, PJI after the index surgery, prosthesis nonreimplantation due to ongoing infection, or amputation of the affected limb. The authors noted that patients infected with *S. aureus* were more likely to fail than those infected with *S. epidermidis*. Interestingly, SCVs were not associated with excess treatment failure compared to WT infections in their study.

Askar et al. [29] presented a case of a 50-year-old female with a long history of knee problems and several orthopedic/surgical interventions in the affected joint ended with a total knee replacement (TKR). Following the replacement, the patient required two subsequent manipulations. PJI manifested with pain and a draining sinus developed 2 years after the index surgery and 4 months after the second manipulation. The patient underwent a first-stage revision of the TKR during which tissue samples and the explanted prosthetic implants were obtained and sent for the microbiological culture. Growth of *S. lugdunensis* was detected in the tissue samples and the sonicate fluid that yielded growth of both WT and SCVs of *S. lugdunensis*. The two isolates were identified to the species level and shown indistinguishable by MALDI-TOF. Their antibiotic susceptibility profiles were similar. Following the revision surgery, the patient was treated with a 6-week course of intravenous ceftriaxone, teicoplanin, and rifampin. Oral treatment with flucloxacillin and rifampin was continued for the next 6 weeks.

Argemi et al. [30] reported an in interesting case of *S. lugdunensis* prosthetic knee infection that relapsed 1 year later with the same strain that changed from the WT fully susceptible to antibiotics, to the antibiotic-resistant SCV phenotype. The patient was a 70-year-old woman with a 4-year history of a total knee arthroplasty complicated by two episodes of PJI. The first one developed a year after the index surgery and was caused by a multidrug-resistant *S. epidermidis*. Treatment included prosthesis exchange and a 6-week antimicrobial therapy with rifampin and daptomycin. The second episode of PJI due to a fully antibiotic-susceptible *S. lugdunensis* developed 2 years after the *S. epidermidis* PJI episode. All bacteriological samples, synovial tissue, joint fluid, and polyethylene, were positive for *S. lugdunensis* with the same regular colony morphology. Treatment was initially successful, but the patient experienced mechanical joint pain 3 months after antibiotic discontinuation that slowly worsened until a sinus tract with purulent discharge developed. The patient remained afebrile; X-ray of the prosthesis did not reveal prosthesis loosening. Preoperative synovial fluid aspiration was sterile but purulent, and decision was made to perform a one-stage exchange of the prosthesis with the prescription of an empirical antibiotic therapy. Several samples including periprosthetic biopsies, sonicate fluid of the removed material inoculated in pediatric blood culture bottle, and synovial tissue cultures were analyzed. The first positive culture results were obtained from pediatric blood cultures after 3 days of incubation, followed by all samples sub-cultured on sheep blood agar solid media that yielded *S. lugdunensis* SCV. Antibiotic susceptibility testing showed that the strain was resistant to fluoroquinolones and amikacin. The authors speculated that *S. lugdunensis* infection persisted despite initial antibiotic treatment as both WT and SCV strains belonged to the same genetic cluster by clustering with MLST (multilocus sequence typing) scheme, and a conversion to SCV could have been a result of the previous exposure to quinolones or rifampicin. The patient was treated with fusidic acid and rifampicin for three months. The 1-year follow-up showed that the patient fully recovered.

#### Laboratory identification and characterization of CoNS SCVs

Due to fastidious growth characteristics, uncommon physiologic, metabolic features, and frequent instability, SCVs represent a challenge for the laboratory identification and characterization [11, 70]. Their detection is of particular clinical significance as their isolation from patient specimens is indicative of a chronic course of infection and may require specific therapeutic considerations [11].

As discussed previously, CoNS SCVs have been typically associated with foreign-body associated infection. Therefore, their successful cultivation requires the collection of high-quality specimens including joint aspirates, intraoperative tissue samples, and biopsy specimens as well as the sonicate fluid derived from the action of ultrasounds on the implant material in order to remove the biofilm-adherent microbial cells from its surface [11, 71]. Blood samples have also been reported valuable, especially in patients with pacemaker-related infections [36].

**Fig. 1:**
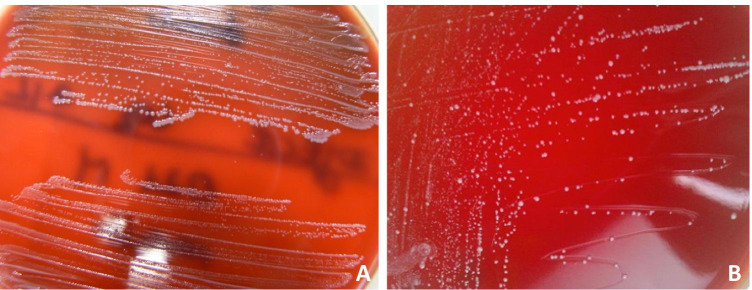
*Staphylococcus epidermidis* culture on Columbia blood agar. **a** 48-h culture of the clinical *S. epidermidis* (isolated from a patient with PJI) demonstrating normal phenotype (top) and SCV morphology (bottom). **b** 72-h culture of the SCV (dynamic/heterogenous) *S. epidermidis* strain isolated from a patient with PJI (unpublished results)

It should be kept in mind that SCVs typically produce non-pigmented, nonhemolytic pinpoint colonies that is about 1/10 the size of the parental WT strain. Thymidine-dependent SCVs frequently exhibit an unusual morphology of colonies resembling “fried eggs” and are characterized by translucent edges surrounding a smaller, elevated pigmented center [11, 45]. Growth of SCVs is observed after 48–72 h of incubation on blood agar plates. Moreover, SCVs can be suspected if they produce very small colonies on Columbia agar but grow nearly normally on Schaedler agar which contains hemin (with CO_2_ or anaerobically) [11, 36, 45] (Fig. 1a and b).

Conventional biochemical procedures used for bacterial identification are of a limited value in case of SCVs, mainly due to their anaerobic metabolism and a reduced carbon flux through the citric acid cycle. These metabolic changes combined with a slow growth account for a lack or at least reduction of biochemical reactions which leads to non- or misidentification in the classical extensive scheme for fermentation, oxidation, hydrolysis, and degradation of chemical substrates but also for commercially available identification systems [11]. Hence, alternative identification approaches have been employed and used with increasing frequency including MALDI-TOF MS analysis (with particular attention paid to the amount of colonial material that should be equal to the amount applied for WT staphylococci) and molecular assays such as the PCR and sequencing [11, 45]. It is worth mentioning that in the majority of literature reports on clinical CoNS SCVs, their laboratory identification was confirmed using the aforementioned techniques due to frequent problems with biochemical identification [29–36] (Table 1).

**Fig. 2:**
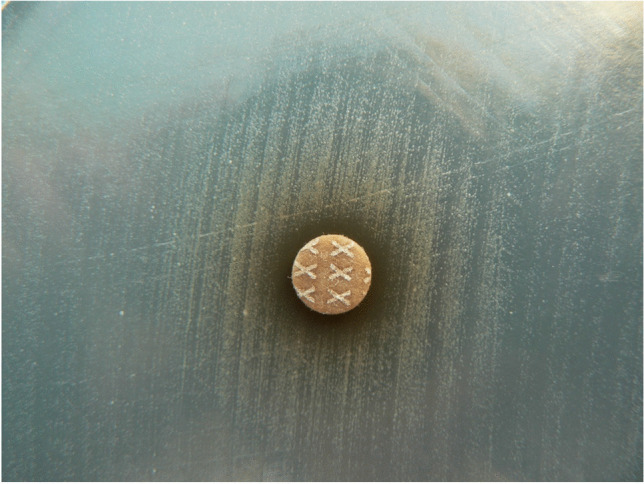
Hemin auxotrophy of the clinical *Staphylococcus epidermidis* SCV strain isolated from a patient with PJI (Mueller–Hinton agar)

Determination of auxotrophy of staphylococcal SCVs is one of the most important tests used for their characterization and understanding of mechanisms underlying the selection of this phenotype [11]. Auxotrophy testing for hemin, menadione, and thymidine can be tested by the application of discs impregnated with the relevant solutions of these compounds to the top of the solid agar plate containing a chemically defined medium inoculated with the tested isolate and has been described in detail by Kahl [11]. The isolate is considered auxotrophic if it shows increased growth around the impregnated disc compared to the periphery [33] (Fig. 2).

Auxotrophy testing among clinically derived CoNS SCVs has revealed variable results including identification of strains with no defined dependence [31, 33], CO_2_ dependence [31, 33], hemin- or menadione-auxotrophy [31, 32], and combined auxotrophism (CO_2_ + menadione [31]; hemin + menadione [32]; hemin + menadione + thymidine [28]).

#### Antibiotic activity against SCVs

Therapy of infections in which staphylococcal SCVs are involved represents a challenge. Metabolic defects observed in SCVs alter their susceptibility to antibiotics, which combined with the ability to survive intracellularly, and for some strains, to produce biofilms, contributes to therapeutic problems [72]. The decreased growth of SCVs may also affect the efficacy of antibiotics, particularly those that are active against dividing microorganisms [11]. Moreover, it should be remembered that under conditions that hamper the achievement of high antibiotic concentrations including bone sequestra or malperfusion of inflamed or necrotic tissue areas, the bacteria are exposed to low or sub-inhibitory antibiotic concentrations which may actually favor the selection of SCVs [11].

Although Tande et al. [31] reported in their study that SCVs including those produced by CoNS were not associated with excess PJI treatment failure compared to the WT infections, the abovementioned properties of SCV morphotype may account for difficulties in bacterial eradication.

There have been several studies addressing the potential activity of antimicrobial agents against CoNS SCVs.

Wu et al. [73] assessed daptomycin and vancomycin pharmacodynamics against a site-directed *hemB* mutant of *S. epidermidis* displaying the SCV phenotype compared to its parental WT and showed that the maximal killing effect decreased by 7.7-fold for vancomycin and 1.5-fold for daptomycin against the SCV mutant. The authors suggested that the decreased activity of vancomycin against the *hemB* mutant could have been associated, in part, with the strain’s enhanced adhesive properties, according to previous reports on increased amounts of polysaccharide intercellular adhesin and stronger adhesion properties exhibited by *hemB* mutants [64]. They linked increased adhesion with potentially enhanced biofilm properties and cell wall sequestration mechanisms hampering antimicrobial activity. Results obtained by Wu et al. [73] were consistent with earlier studies of *hemB* mutants of *S. aureus* where vancomycin failed to achieve bactericidal activity [74]. In contrast to vancomycin, daptomycin retained much of its activity against the SCV strain. The results of Wu et al. [73] were supported by the study in which it was confirmed that the bactericidal activity of daptomycin against *S. aureus* does not require cell division or active metabolism [75].

It has been observed that, in vitro, aminoglycosides and antifolate agents show high MICs for electron-transport-defective and thymidine-dependent SCVs, respectively. The other antibiotic classes usually show MICs comparable to those measured for their WT counterparts, but they demonstrate less bactericidal activity [72].

Maduka-Ezeh et al. [33] found that five out of the 11 *S. epidermidis* SCVs, demonstrated resistance to at least one aminoglycoside tested, nine were *mecA* PCR positive and one was TMP-SXT resistant. Among three SCV-WT pairs, only one SCV demonstrated less aminoglycoside and TMP-SXT susceptibility than its WT. The authors suggested that aminoglycoside resistance may not be a uniform property of SCVs but may be limited to SCVs of certain species and/or to SCVs demonstrating hemin or menadione dependence as none of their isolates demonstrated this type of auxotrophy. Indeed, in the study of Bogut et al. [32], in which seven SCVs were hemin-dependent, and one isolate demonstrated a combined hemin and menadione dependence, all but one isolates were gentamicin resistant. The TMP-SXT resistance was observed less frequently and was reported for only one SCV strain. Moreover, for 3 out of 5 and 2 out of 5 clonal SCV-WT pairs, SCVs had higher gentamicin and TMP-SXT MIC values, respectively, than their WT counterparts even when they remained within the same resistance/susceptibility range.

Idelevich et al. [76] tested 31 *S. aureus* clinical SCV-NP strain pairs encompassing 28 MSSA and 3 MRSA pairs as well as four pairs of CoNS strains comprising two *S. epidermidis* SCV-NP pairs (both methicillin resistant), one methicillin-susceptible *Staphylococcus capitis* SCV-NP pair, and one methicillin-susceptible *Staphylococcus lugdunensis* SCV-NP pair. The study showed that ciprofloxacin MICs were higher for SCVs than for the WTs, while no marked difference was observed for other fluoroquinolones, including moxifloxacin, levofloxacin, and finafloxacin. Of note, the authors revealed the enhanced activity of finafloxacin at low pH regardless of the bacterial phenotype, which might facilitate SCV eradication in acidic environments such as foci of osteomyelitis, skin infections, abscesses, and lung infections in patients with CF.

SCVs also represent a challenge in terms of antibiotic susceptibility testing. Slow growth of SCVs and their reduced metabolism make the results of all time- and density-influenced methods of susceptibility testing difficult or impossible to interpret [11].

Therefore, the results of the disc diffusion or automated overnight methods are usually invalid since the SCV growth cannot be seen on the agar or detected by optical density measurements in automated systems. Moreover, clinical isolates are often a mixed population of parent strains and SCVs. Therefore, even a small percentage of the WT organisms can rapidly replace the SCVs in liquid medium in an overnight culture due to a significantly longer doubling time of SCVs. Therefore, the SCVs may be overgrown to such an extent that they may not be included in the inoculum used for susceptibility testing. Moreover, instability of many clinical SCVs is another potential reason for not including them in susceptibility testing [37, 70].

Proctor et al. [37] points that in addition to limitations associated with slow growth of SCVs, selection of media for susceptibility testing is also important. Some batches of the Mueller–Hinton agar may contain concentrations of thymidine high enough to cause reversion of an SCV to the parental phenotype that is more susceptible to sulfonamide antimicrobial agents. More enriched media such as the brain–heart infusion medium, in turn, contain relatively large amounts of menadione, which will relieve the SCV phenotype of menadione-dependent isolates.

Apart from growth rate-related limitations, antibiotic susceptibility testing of SCVs using broth or agar dilution methods has been considered a practice of the best validity [11, 37].

Simultaneous testing of a co-isolated WT strain whose clonality with the SCV has been confirmed by molecular methods (e.g., PFGE, MLST) is also advisable. Comparing isogenic pairs of the WT and SCV phenotypes may show reduced susceptibilities of the latter. On the other hand, similar categorization in terms of resistance for the two phenotypes has also been reported [11]. Tande et al. [31] reported that while the majority of SCVs were clonally related to the normal phenotype bacteria, up to one-third had discordant susceptibilities using Etest or disc diffusion method. This observation highlights the need for performing susceptibility testing on all bacterial morphotypes.

Several authors have used a disc diffusion methodology or E-tests on the Mueller–Hinton (M-H) agar extending the incubation period to 48 h in order to accommodate the slow growth of SCV [31–33]. According to Maduka-Ezeh et al. [33], if the susceptibility testing results of control strains are within the limits, the results can be considered reliable. Incubation with CO_2_ can also support bacterial growth [11]. However, the readout of the plates is often difficult and sometimes does not provide clear results for all antimicrobial drugs tested [11]. Tande et al. [31], for example, used the M-H agar supplemented with 5% sheep blood due to poor SCV growth on the M-H agar. Nevertheless, interpretation of the susceptibility results for trimethoprim-sulfamethoxazole using E-test was difficult or impossible for some of the isolates due to poor growth and indistinct boundaries, even with blood-containing medium [31].

For determination of methicillin resistance in SCVs, recommended methods include the PCR or other molecular detection of the *mecA*/*mecC* gene and an anti-PBP2a slide agglutination assay modified by a drastically increased number of bacterial colonies [11, 45]. Errors may occur when these variants are resistant to oxacillin when tested by disc-diffusion test, E-test, microdilution test, and automated susceptibility-testing systems as well as anti-PBP2a slide-latex agglutination tests [45]. Discrepancies regarding the phenotypically expressed susceptibility to oxacillin have also been observed [31–33]. Oxacillin has been considered one of the most frequent antimicrobial agents with discordant susceptibilities reported in several studies for cultured SCV-WT pairs. Tande et al. [31] reported fluctuation of cefoxitin disc diameter complicating the interpretation in two *mecA*-positive isolates. Maduka-Ezeh et al. [33] reported phenotypic oxacillin resistance detected by agar dilution and disc diffusion with cefoxitin in 8 out of 9 *mecA*-positive *S. epidermidis* isolates. Similarly, Bogut et al. [32] detected phenotypic resistance using cefoxitin disc in 6 out of 7 *mecA*-positive SCVs. This may suggest that the *mecA* may not be expressed in some isolates, possibly as a result of repressor mechanisms which have been previously described for *S. aureus* [33]. On the other hand, phenotypic oxacillin resistance may depend on factors other than the presence or absence of *mecA* [31].

### Pathomechanism of the S. epidermidis SCV infections

The success of *S. epidermidis* as a pathogen depends on its ability to flexibly adapt to environmental stresses, such as antimicrobial treatment, limited oxygen, or temperature fluctuations [77]. Bacteria can quickly respond to unfavorable environmental or stressful conditions by undergoing dynamic evolutionary changes and switch to SCV mode of growth which can provide a strong advantage and promote their survival [78]. This capacity enables bacteria to persist intracellularly, entering a stage of dormancy, and consequently promoting a chronic course of infection. Indeed, several in vitro and in vivo studies argue that coagulase negative staphylococci, such as *S. epidermidis* can invade and persist for varying lengths of time in many non-professional phagocytic cells, such as epithelial cells, endothelial cells, fibroblasts, and osteoblasts and even within professional phagocytes such as human monocyte-derived macrophages [79–81]. The intracellular stage may be thus crucial for persistence, dissemination, and serve as a potential source for recurrent infection [82, 83].

#### Increased adherence and uptake

For SCVs, attachment is a critical first step that precedes their internalization within host cells [13, 15]. Typically, staphylococci express a wide variety of host factor binding proteins involved in their adhesion. The interaction between *S. aureus* and host cells mainly involves fibronectin (Fn) which bridges the α_5_β_1_ integrin in mammalian cells and a number of bacterial adhesins, including Fn binding proteins (FnBPs).

In comparison with *S. aureus*, less is known about the surface proteins involved in *S. epidermidis* attachment. Nevertheless, recently the staphylococcal surface-associated autolysin, AtlE has been suggested to possess adhesive functions, besides its primary role in cell separation, and directly interact with the heat shock cognate protein 70 (Hsc70) as host cell receptor [84]. Besides autolisin/adhesins, the integrin α_5_β_1_ and Fn seem to be important for internalization of *S. epidermidis*, similar to FnBPs-mediated *S. aureus* uptake [85, 86]. Schlesier et al. [85] suggest that as Atl is ubiquitous among all staphylococcal species, it may therefore represent the good substitution for the FnBPs in staphylococci lacking FnBP. In the model they propose, the interaction between *S. epidermidis* and host cells is believed to occur through a bridging mechanism, in which bacterial AtlE and Fn bridges to the α_5_β_1_ integrin and Hsc70 as a coreceptor [85].

Irrespective of the phenotype, CoNS SCVs utilize the same adhesion and invasion mechanisms as WT microorganisms, but most importantly, SCVs are much more efficient in these processes than their progenitors which facilitate their interaction with the host [87].

Professional and non-professional phagocytes constitute a niche for bacteria with an intracellular lifestyle. Once attached to the host cell, SCVs, like their WT counterparts, induce host-cell changes by actin rearrangement, which mediates internalization and leads to the activation of signaling pathways of the src family tyrosine kinases [82, 87, 88]. Invasion signaling further involves the phosphatidylinositol 3-kinase (PI3K) pathway, the effector molecule of which is the Akt kinase.

Accumulated evidence shows that Akt activity correlates with the internalization of persistent pathogens such as *Mycobacterium tuberculosis*, *Chlamydia pneumoniae*, *Helicobacter pylori* and latent viruses, including HIV and herpes viruses, suggesting that this kinase might be a central host factor targeted by pathogens to take control over their phagocytosis and modulate the host immune response [82, 89–91]. SCV strains of *S. epidermidis* can also manipulate this signaling pathway by the activation of Akt in macrophages [92]. It was also recorded that SCV strains are able to activate Akt, even in the presence of its inhibitor, wortmannin. This suggests that the PI3K/Akt pathway is an important element used by SCV for internalization and intracellular survival. Bacteria may stimulate Akt phosphorylation even in the presence of a PI3K inhibitor, probably by using other possible ways whose activity is not altered by wortmannin [92]. The same signaling pathway is also involved in internalization of *S. epidermidis* in vascular endothelial cells [85], suggesting that similar mechanism is common for phagocytic and non-phagocytic cells. As there is currently a little understanding of the molecular mechanisms that lead to internalization of CoNS, the signaling events mediated by Src and PI3K/Akt pathway may represent the first or even sole internalization mechanism adopted by *S. epidermidis* and other CoNS, both WT and SCVs [85].

#### Intracellular persistence

After cellular invasion, intracellular persistence of bacteria can occur. Although considered as a non-invasive extracellular bacterium, *S. epidermidis* appears to be well adapted to the intracellular milieu of eukaryotic cells and thus might be termed a facultative intracellular pathogen [13, 88, 93, 94]. From a clinical standpoint, this intracellular stage helps to account for chronic, relapsing infections that are refractory to antimicrobial therapy [95]. In addition, their presence in macrophages may indicate the effectiveness of such a strategy in efficient spreading of the pathogen to areas distant from the primary site of infection [82, 87].

**Fig. 3:**
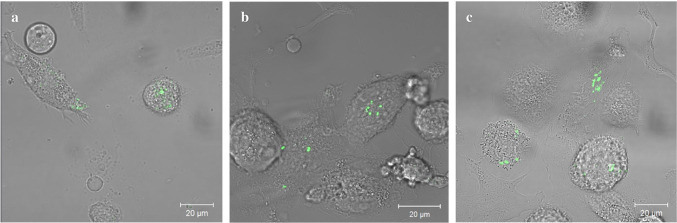
Intracellularly persisting SCVs of *S. epidermidis* analyzed by confocal microscopy*.* FITC-labelled bacteria (green) in macrophages **a** 2 h post phagocytosis (p.p.); **b** 24 h p.p. **c** 72 h p.p. Scale bar 20 µm

The phagocytosis is one of the most important mechanisms for the elimination of infectious bacteria [5]. Professional phagocytes, such as neutrophils, macrophages, and dendritic cells, are design to kill invading microorganisms with a combination of various microbicidal systems [93]. However, some pathogenic organisms have developed mechanisms to resist the phagocytosis. They represent very successful pathogens, such as *Mycobacterium tuberculosis*. Surprisingly, apart from typical intracellular pathogens, *S. epidermidis* SCVs have developed strategies to evade a bactericidal attack inside macrophages. The in vitro studies confirmed that *S. epidermidis* SCVs can survive inside macrophages and neutrophils for at least 3 days without significant alteration in cells’ viability (Fig. 3). Moreover, SCVs proliferate intracellularly more successfully than their counterparts. Several research groups indicated that although the number of intracellular SCVs shortly after internalization (2 h) was lower in comparison to the WT strains, their counts was barely reduced during 24 h of the study time, unlike the parental strains as their numbers significantly decreased within the first 24 h post phagocytosis [96, 97]. Yet, the persisting SCVs can escape from their intracellular shelter into extracellular milieu giving rise to recurrent infection. This observation is indeed supported by clinical observations in which infections caused by SCVs phenotypes persisted asymptomatically for many years, until the time when certain conditions of the patient disturbed the balance between the bacteria and the host response leading to a new acute episode of an infection [11, 98, 99].

Bacterial SCVs can also persist within non-professional phagocytes, which are believed to be the main pathogenicity mechanism leading to chronic infection [16, 81]. They possess mechanisms similar to those used by macrophages to phagocytose and degrade microorganisms. However, due to the attenuated virulence associated with the SCV phenotype, they can persist within morphologically intact host cells for long periods of time [17, 100].

Monitoring bacterial internalization provides useful information about their virulence and fate inside host cells. It is now widely accepted that intracellular bacteria, in order to survive, use one of three strategies: they modify the process of phagolysosome formation, adapt to the low pH of phagolysosomes or escape from phagolysosomes into the cytoplasm [95, 101].

**Fig. 4:**
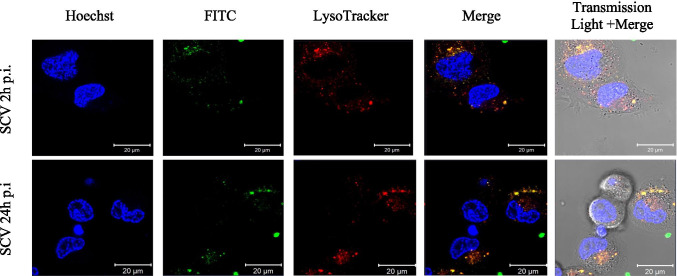
Intracellular colocalization of *Staphylococcus epidermidis SCV* within macrophages. The intracellular localization of persisting bacteria (FITC; green) analyzed by confocal microscopy. Lysosomes are visualized with LysoTracker Red (red) and nuclei are stained with Hoechst (blue). Scale bar 20 μm

When intracellular trafficking of the bacteria was monitored through confocal studies with an acidotrophic probe LysoTracker, indicator of phagosome pH, occupying compartments that are consistent with features of late endosomes and lysosomes, it was found that intracellular survival within an acidic niche of phagolysosomes is indeed possible (Fig. 4). On the other hand, phagosomes containing WT bacteria showed a dramatically decreased level of colocalization over the period of 24 h [82, 102]. The ability to persist inside phagolysosomes is probably connected with decreased transmembrane potential or altered cell wall composition of SCVs [11, 82, 102]. Moreover, it was shown that the SCV formation is triggered by harsh conditions, such as exposure to acidic environment, which further increases the probability of their survival [11, 82].

#### Evading the host’s protective mechanisms

Adaptation to the intracellular lifestyle provides various advantages for SCVs: it ensures protection against the host’s humoral immune response and makes it less accessible to antibiotics [101]. However, the infected host cell possesses defense mechanisms which specifically target these microorganisms. Persisting bacterial pathogens have therefore developed different strategies to successfully evade the host innate response and establish an intracellular infection. To survive inside the hostile intracellular environment, SCVs manipulate the host cellular signaling pathways to avoid activation of the host immune response or cytotoxic effects in order to create an immune tolerant environment [11, 77].

Activated macrophages provide an inhospitable environment for most microorganisms, and the phagolysosome is the definitive antimicrobial organelle [103]. Apart from the acidic environment, nitric oxide, produced by inducible NO synthase (iNOS), is classified as one of their main bactericidal mechanisms [103]. iNOS is undetectable in the resting phagocytes and is only expressed in response to a variety of immunological and inflammatory stimuli. When iNOS is upregulated, abundant NO is produced for long periods, reacting with proteins and the DNA of ingested microorganisms. Also, it can directly impair bacterial enzymes and affect microbial growth [103]. But despite the generation of NO, and other microbicidals, it is not surprising that many successful intracellular pathogens have evolved multiple strategies to resist phagolysosome contents [103]. Of note, *S. epidermidis* SCVs induce iNOS synthesis less efficiently when compared to their progenitors [102]. As mentioned before, a reduced transmembrane potential can protect SCVs form bactericidal cationic proteins produced by neutrophils, macrophages, and epithelial cells [15, 104]. But also, by using certain amino acids, they can block the activity of key host enzymes involved in the immune response, such as iNOS [15, 105]. Small colony variants of staphylococci were also found to be resistant to other innate host defense peptides, such as lactoferricin B and protamine. But the exact mechanism of the resistance is still unknown. It is postulated, however, that as SCVs characteristically have an altered metabolic state and lactoferricin B is effective only against metabolically active bacteria, the antimicrobial effectiveness of the peptide is limited [106].

Regulation of the immune response to bacterial infections as well as the intracellular fate of the invading pathogen relies significantly on cytokines secretion. Of note, because of its commensal nature, cytokine release induced by *S. epidermidis* has rarely been studied. The observations made by Megyeri et al. indicate, however, that commensal *S. epidermidis* induces lower levels of pro-inflammatory cytokines compared to *S. aureus* [107].

When macrophages are exposed to inflammatory stimuli, TNF-α is one of the first to be released [108]. This potent cytokine stimulates the acute phase of the inflammatory response by triggering the localized accumulation of leukocytes and inflammatory mediators. TNF-α also increases the phagocytic ability of macrophages and enhances the killing of the pathogen, particularly in concert with interferon γ, leading to the eradication of an infectious agent [108, 109]. In vitro stimulation of macrophages with staphylococcal WT and SCV strains leads to a rapid release of pro-inflammatory cytokines, such as TNF-α, IL-6, and IL-8, but SCVs showed decreased cytokine production when compared to WT counterparts [96, 109].

Professional intracellular pathogens can manipulate cytokine secretion to facilitate their persistence during infection and IL-10 seems to play a detrimental role in this evasion strategy [89]. The cytokine is a potent anti-inflammatory mediator, suppressing macrophage activation and the production of pro-inflammatory cytokines [109]. Macrophages exposed to this cytokine lower their microbicidal activity preventing the stimulation of an appropriate proinflammatory response required for bacterial neutralization, thereby creating a microenvironment in which it can persist [109].

SCVs can manipulate macrophage production of IL-10, as they induce significantly more cytokine synthesis from macrophages when compared to WT strains [96]. This finding confirms that bacterial SCVs by stimulating IL-10 release and preventing excessive inflammation establish a persistent intracellular infection.

Available data suggest that lower TNF-α level together with high IL-10 synthesis can be associated with chronicity found in staphylococcal SCV biomaterial-associated infections. Indeed, during persistence, the SCV largely avoid activation of the host immune response. This sub-acute nature of *S. epidermidis* infections can partially explain why chronic infections with persistent staphylococcal SCVs often have a low inflammatory character.

## Conclusions and perspectives

CoNS infections are of an increasing burden to the healthcare system mainly due to a widespread use of implanted medical devices. Biofilm formation coupled with a significant phenotypic variability and intracellular persistence as well as the consequent diagnostic and therapeutic challenges contributes to their increasing clinical significance.

The results of this literature review highlight how intracellular survival and phenotypic switching defines a powerful immune escape mechanism and an alternative route to explain a chronic infection caused by CoNS, especially *S. epidermidis.* Understanding staphylococcal survival strategies and the immune mechanisms that result in killing of intracellular pathogens will deepen our insight into the pathogenesis of chronic and therapy-refractive infections. This has also an important clinical implication. Targeting the bacterial adaptation strategies should be considered as a novel therapeutic and preventative strategy to avoid the formation of SCVs and the development of chronic infection as well as to enhance complete bacterial eradication.

## Data Availability

Not applicable.
